# Rewriting Inflammation in IBD: Lipidomics from Pathogenesis to Clinical Application

**DOI:** 10.3390/microorganisms14071432

**Published:** 2026-06-30

**Authors:** Christopher Patteril, Chiara Pezzella, Pierluigi Puca, Federica Di Vincenzo, Loris Riccardo Lopetuso, Lucrezia Laterza, Daniele Napolitano, Giovanni Cammarota, Alfredo Papa, Antonio Gasbarrini, Franco Scaldaferri

**Affiliations:** 1 Dipartimento di Medicina e Chirurgia Traslazionale, Università Cattolica del Sacro Cuore, 00168 Rome, Italy; christopher.patteril01@icatt.it (C.P.); pierluigi.puca@unicatt.it (P.P.); federica.divincenzo01@icatt.it (F.D.V.); alfredo.papa@unicatt.it (A.P.);; 2 UOC Gastroenterologia, Fondazione Policlinico Universitario Agostino Gemelli IRCCS, 00168 Rome, Italy; 3 IBD Unit, Centro per le Malattie dell’Apparato Digerente (CEMAD), Dipartimento di Scienze Mediche e Chirurgiche, Fondazione Policlinico Universitario Agostino Gemelli IRCCS, 00168 Rome, Italy; 4 Department of Life Science, Health, and Health Professions, Link Campus University, 00165 Rome, Italy; 5 SITRA-Scientific Direction, Fondazione Policlinico Universitario Agostino Gemelli IRCCS, 00168 Rome, Italy; 6 Centro per le Malattie dell’Apparato Digerente (CEMAD), Dipartimento di Scienze Mediche e Chirurgiche, Fondazione Policlinico Universitario Agostino Gemelli IRCCS, 00168 Rome, Italy

**Keywords:** inflammatory bowel disease, Crohn disease, ulcerative colitis, lipidomics, biomarkers, sphingolipids, bile acids and salts, mass spectrometry

## Abstract

Lipids (sphingolipids, fatty acids, phospholipids, and lipoproteins) are vital to intestinal barrier integrity, as precursors for pro-inflammatory and pro-resolving mediators and undergo remodelling through host microbiome interactions. Accumulating evidence implicates the Western diet—high in long-chain saturated and omega-6 polyunsaturated fatty acids and low in omega-3—in both the onset and progression of IBD. In contrast, microbiota derived lipid metabolites, including short-chain fatty acids and secondary bile acids, contribute to mucosal homeostasis and immune regulation. This review is structured around three interconnected pillars. First, we classified lipidomic alterations in IBD across major lipid classes: sphingolipids, fatty acids, phospholipids, and lipoproteins by integrating host, dietary, and microbiome contributions. Second, we examined the potential of lipidomics in IBD as a source of prognostic, diagnostic and therapy response biomarkers. Third, we critically assessed the challenges that currently limit clinical implementation including analytical variability, pre-analytical confounding, small cohort sizes, and the lack of prospective validation. Addressing these barriers will be essential to fully realise the potential of lipidomics in advancing personalised care for patients with IBD.

## 1. Introduction

Inflammatory bowel disease (IBD) encompasses a group of chronic, immune-mediated conditions affecting the gastrointestinal tract, including Crohn’s disease (CD), ulcerative colitis (UC), and less-defined entities such as IBD-unclassified (IBD-U) and indeterminate colitis [1,2]. Both CD and UC share a multifactorial pathogenesis rooted in the interaction between genetic predisposition, environmental exposures, gut microbiota composition, and immune dysregulation, ultimately producing a state of unresolved mucosal inflammation and tissue injury [1,2].

Although several medications are now available, IBD diagnosis relies on a composite of clinical, biochemical and imaging parameters while standardised molecular biomarkers are still lacking. Omics technologies offer a systematic means of characterising the molecular landscape of disease at scale [3,4]. While genetic and transcriptomic studies have been explored extensively in IBD, lipidomics seems to capture the dynamic interplay between metabolism, immunity, and the microbiome. Lipidomics employs mass spectrometry to systematically profile lipid species and their associated metabolic pathways, using three principal analytical approaches: targeted lipidomics, which quantifies predefined lipid classes using high-sensitivity instruments such as triple-quadrupole mass spectrometers; shotgun lipidomics, in which complex lipid extracts are analysed directly, preferably on high-resolution platforms such as Orbitrap instruments, without prior chromatographic separation; imaging lipidomics, which maps lipid distributions across tissue sections through surface ionisation [4].

Lipids constitute structural components of the intestinal barrier, serve as precursors to pro-inflammatory and pro-resolving mediators, and are subject to extensive remodelling by the gut microbiota. Emerging evidence links the Western dietary lipid pattern to IBD risk and progression, a relationship developed mechanistically in Section 3.6 [5,6]. Whereas specific microbially derived lipid metabolites such as short-chain fatty acids (SCFAs) and secondary bile acids, exert protective effects on mucosal homeostasis [7,8].

The implementation of lipidomics into the understanding of IBD pathogenesis could shed new light on traditional IBD classification. Beyond cataloguing these associations, we argue that lipidomic data may ultimately support a biochemical reclassification of IBD—defining mechanistic endotypes that cut across the conventional Crohn’s disease and ulcerative colitis division. We develop this framework in the Discussion, while delineating the validation it would require before it could inform clinical practice. This could lead to an improved comprehension of the disease that could help define disease trajectories, predict response to therapies and favour more personalised medicine.

This review aims to draw attention to lipidomics as an underexplored yet promising field in IBD research, given the growing evidence implicating lipids and their associated pathways in disease pathogenesis, including their potential role in disease discrimination, monitoring of mucosal inflammation, prediction of therapeutic response, and refinement of treat-to-target strategies.

## 2. Materials and Methods

This work is a structured narrative review rather than a systematic review; it was designed to deliver a critical, mechanistically organised synthesis of lipidomics in IBD rather than an exhaustive enumeration of every primary study. A structured search was performed in PubMed/MEDLINE, Embase, Scopus, and Web of Science for articles published between January 2002 and December 2025, supplemented by landmark older references where foundational. Search terms combined controlled-vocabulary and free-text terms for inflammatory bowel disease, ulcerative colitis, and Crohn’s disease with terms for lipidomics and the individual lipid classes discussed herein (sphingolipids, glycerophospholipids, glycolipids, glycerolipids, fatty acids, and lipoproteins), as well as specialised pro-resolving mediators, bile acids, biomarkers, and therapeutic response. Original human studies, multi-cohort validation studies, Mendelian randomisation analyses, and mechanistic work in validated murine colitis models were prioritised; narrative and systematic reviews were cited only when they synthesised primary evidence not otherwise available, and methodological papers on mass-spectrometry lipidomics, pre-analytical variability, and biomarker reproducibility were included to frame the translational barriers discussed in Section 5 and Section 7. Studies were excluded if not published in English, if they lacked human or IBD-relevant preclinical data, or if they did not report lipid-specific measurements. Reference lists of included articles were hand-searched for additional citations. Study selection and synthesis were guided by topical relevance and expert appraisal rather than by formal systematic screening or dual independent review; accordingly, the absence of a PRISMA-based selection process and formal risk-of-bias assessment is acknowledged as a limitation, and the cited literature should be read as a representative critical synthesis.

## 3. Alterations in Lipid Profiles in IBD

Lipids are a structurally and functionally heterogeneous class of hydrophobic molecules. In intestinal physiology, they cover a broad spectrum of bioactive species, including dietary-derived fatty acids, microbially synthesised sphingolipids, and phospholipid-derived signalling mediators, each of which may be uniquely involved in the pathogenesis and progression of IBD [4,9,10,11].

### 3.1. Sphingolipids

Sphingolipids are fundamental constituents of the cell membrane architecture and include two broad subclasses: complex sphingolipids such as sphingomyelin, cerebrosides, and gangliosides; and simple sphingolipids such as ceramide and sphingosine, together with their phosphorylated forms, sphingosine-1-phosphate (S1P) and ceramide-1-phosphate (C1P), which are pivotal regulators of cell survival, apoptosis, and inflammatory signalling [10,11].

The relationship between sphingolipid species and IBD activity has been characterised through correlation with established inflammatory markers. Cer 18:1;O2/24:0 and HexCer 18:1;O2/22:0, 23:0, and 24:0 are inversely correlated with both C-reactive protein (CRP) and faecal calprotectin. Ceramide 18:1;O2/26:0 shows a negative correlation with CRP alone while ceramide 18:1;O2/23:0 and 18:1;O2/26:0 do not associate with calprotectin, suggesting that distinct ceramide species track different dimensions of mucosal and systemic inflammation [10].

Elger et al. demonstrated that an elevated ratio of long-chain (LC) to very-long-chain ceramides (VLC) in serum is associated with disease severity in IBD and primary sclerosing cholangitis (PSC). Notably, this LC/VLC ceramide ratio is elevated in IBD, PSC-IBD, and PSC compared to healthy controls thus establishing it as a possible quantitative index of disease severity [10].

The pattern of sphingolipid alteration differs substantially according to treatment status and disease subtype. Moe et al. characterised the mucosal lipid profile across treatment-refractory and treatment-naïve UC patients and demonstrated that refractory UC patients exhibit pronounced decreases in ceramides, hexosylceramides, sphingomyelins, glycerophosphocholines (PC), glycerophosphoethanolamines (PE), and glycerophosphoserines (PS) relative to both treatment-naïve patients and healthy controls [11].However, treatment-naïve UC patients show elevated ceramide and sphingomyelin levels compared to controls, whereas remission profiles approach those of healthy controls [11]. In CD, refractory patients showed major alterations in PE, SM, PS, HexCer, and PC compared to controls, but no consistent differences emerged between treatment-naïve CD patients and controls, nor between refractory and naïve CD patients, a pattern that may reflect the greater heterogeneity in CD location and transmural extent [11].

### 3.2. Glycolipids

Glycolipids comprise glycosphingolipids, glucosyl and galactosylceramides, lactosylceramide, and the sialylated gangliosides together with glyceroglycolipids. Glucosylceramide metabolism is tied to mucosal immune regulation [12]. In IBD patients, UDP-glucose ceramide glucosyltransferase (UGCG) the glycosphingolipid-synthesising enzyme is downregulated, and this loss is reproduced in dextran sulphate sodium (DSS) colitis, with reduced UGCG and glucosylceramide content in mesenteric lymph nodes and splenic T cells [12]. The downstream consequences for the regulatory T cell compartment, and the protective effect of glucosylceramide repletion, are preclinical (murine) and are developed alongside the immune-homeostasis literature in Section 4.2; glucosylceramide thus behaves as a regulator of mucosal tolerance rather than a passive membrane constituent.

The same enzymatic node likely links inflammation to neoplastic progression. Disrupted sphingolipid metabolism promotes inflammation in IBD and fosters a microenvironment permissive to colorectal cancer, with glucosylceramide synthase activity and high tumoural UGCG expression associated with worse outcomes in established disease [13]. Dedicated glycolipid profiling in human IBD cohorts remains limited relative to the phospholipid and sphingolipid literature, and most available data derives from animal models or from glycosphingolipid species captured incidentally within broader lipidomic panels. This gap could be closed by targeted glycosphingolipid lipidomics, including ganglioside and hexosylceramide speciation, thus establishing whether the depletion seen in refractory disease is cause or consequence of impaired barrier and immune function.

### 3.3. Glycerolipids (Triglycerides)

Glycerolipids, mono-, di-, and triacylglycerols are the principal neutral and storage lipids of the lipidome [14]. Unlike the sphingolipid and phospholipid classes, triglycerides show no clear quantitative signature in IBD: a systematic review and meta-analysis of 53 studies did not identify triglycerides among the serum lipid fractions consistently altered in IBD relative to healthy controls, with findings varying by disease activity, nutritional status, and treatment across cohorts [14]. This heterogeneity indicates that the total triglyceride pool is a poor discriminator of disease state, and that any informative glycerolipid signal is more likely to reside at the level of individual molecular species than in bulk concentration [14].

Species-resolved genetic approaches support this. In a bidirectional two-sample Mendelian randomisation analysis of the plasma lipidome, diacylglycerol was causally associated with an increased risk of Crohn’s disease (OR 1.21), placing a specific glycerolipid intermediate, rather than total triglyceride, within the mechanism of the disease [15]. Diacylglycerols occupy a branch point between triglyceride storage and phospholipid synthesis and act as second messengers in protein kinase C signalling [15], offering a plausible route by which glycerolipid flux could shape epithelial and immune responses. Beyond this genetic signal, dedicated acylglycerol lipidomics in IBD tissue remains sparse as most triglyceride data derives from routine serum measurement rather than untargeted profiling. Future studies would benefit from studying species-level glycerolipid remodelling and its relationship to the mucosal phospholipid and sphingolipid shifts.

### 3.4. Phospholipids

Phospholipids are major structural components of the intestinal epithelial membrane and play active roles in barrier function, vesicle trafficking, and PI3K-dependent signalling [16]. In CD, phosphatidylinositol abnormalities are observed with relative preservation of ceramide, phosphatidylcholine, and phosphatidylserine composition, indicating selective phospholipid dysregulation affecting specific intracellular signalling pathways [17]. Intestinal phospholipid disequilibrium can initiate endoplasmic reticulum (ER) stress responses that drive goblet cell necroptosis and spontaneous colitis, as demonstrated in murine models by Kennelly et al. (2021) [16]. Spatial imaging of UC biopsies by MALDI-MSI added another layer. Dysregulated phosphatidylinositols, phosphatidylethanolamines, and PE plasmalogens were found to change in a gradient along the colonic crypt axis, mirroring disrupted colonocyte differentiation. This reveals a phospholipidome that is highly cell-type-specific able to distinguish inflammatory, malignant, and differentiating epithelial states from one another [18].

### 3.5. High-Density Lipoprotein Cholesterol and Lipoprotein Remodelling

High-density lipoprotein cholesterol (HDL-C) is well recognised for its cardiovascular protective and systemic anti-inflammatory properties, and emerging Mendelian randomisation evidence suggests a causal role in IBD susceptibility [19]. Tao et al. demonstrated a causal association between elevated HDL-C levels and a reduced risk of IBD, including both UC and CD, implicating HDL-C in the regulation of mucosal immune homeostasis [19]. The anti-inflammatory mechanism involves interaction with cholesteryl ester transfer protein (CETP): CETP inhibition, which raises HDL-C while reducing transfer of cholesteryl esters to LDL and VLDL, is associated with a reduced risk of developing CD [19].

IBD patients also exhibit a disease activity-dependent compositional shift in LDL particle size from large, atheroprotective subclasses (LDL-1 to LDL-3) toward smaller, denser, more atherogenic species (LDL-4 to LDL-6), while ApoA1 and ApoA2, which are the primary anti-inflammatory constituents of HDL, decline progressively with increasing disease activity [12,20]. These systemic lipoprotein alterations simultaneously position IBD patients at elevated cardiovascular risk independently from conventional lipid fractions.

### 3.6. Fatty Acids and Derived Lipid Mediators

Fatty acids are a class of dietary lipids implicated in immune regulation, gut microbiota modulation, and intestinal barrier maintenance [5,7]. They are classified by carbon chain length into very-long-chain (VLCFAs), long-chain (LCFAs), medium-chain (MCFAs), and short-chain fatty acids (SCFAs), each with distinct immunological profiles [5].

SCFAs, produced by microbial fermentation of dietary fibre in the colon, exert pronounced anti-inflammatory and immunoregulatory effects. They promote CD4+ T cell and innate lymphoid cell (ILC) production of IL-22 through upregulation of the aryl hydrocarbon receptor (AhR) and hypoxia-inducible factor 1-alpha (HIF-1α), thereby maintaining intestinal homeostasis [7]. Among SCFAs, butyrate exerts the most potent anti-inflammatory activity: its interaction with the G protein-coupled receptor GPR109A drives regulatory T cell (Treg) differentiation and IL-10 production [7,8]. The dominant butyrate-producing species, *Eubacterium*, *Faecalibacterium*, and *Roseburia*, belonging to *Clostridium* clusters IV and XIVa of the phylum Firmicutes, are consistently depleted in IBD, with *Faecalibacterium prausnitzii* and the *Clostridium coccoides*/*Eubacterium rectale* group reduced in luminal fractions and *Roseburia* spp. reduced in mucosal fractions of UC samples [8,21].

Long-chain fatty acids (LCFAs) encompass saturated fatty acids (SFAs), monounsaturated fatty acids (MUFAs), and polyunsaturated fatty acids (PUFAs), with divergent immunological effects. Omega-3 PUFAs, derived from eicosapentaenoic acid (EPA) and docosahexaenoic acid (DHA), exert anti-inflammatory effects and modulate the gut microbiota by suppressing *Enterobacteria* and promoting *Bifidobacteria* growth [5]. Omega-6 fatty acids, as precursors of arachidonic acid, have the potential to generate both pro-inflammatory and anti-inflammatory metabolites depending on the enzymatic pathway engaged. An imbalanced omega-6/omega-3 ratio, as characterised by the Western diet, where this ratio may reach approximately 20:1, predisposes to IBD onset and progression [5]. Saturated and trans-unsaturated fatty acids promote inflammation, whereas oleic acid and omega-3 PUFAs display anti-inflammatory actions [5,7,8,21]. Ananthakrishnan et al. confirmed diet as a modifiable risk factor for IBD in a prospective study of women, demonstrating that high dietary intake of long-chain omega-3 PUFAs is associated with a reduced risk of UC, while high trans-unsaturated fat intake is associated with an increased risk [5].

Lipid mediators derived from polyunsaturated fats, including eicosanoids and oxylipins, exert both pro-inflammatory and pro-resolving functions in IBD [22,23,24]. Prostaglandins and leukotrienes derived from arachidonic acid are pro-inflammatory mediators operative at the onset of inflammation, while lipoxin A4 (LXA4), LXB4, and omega-3-derived mediators act as pro-resolving signals during the resolution phase [24]. Masoodi et al. demonstrated in a case–control study that UC colonic mucosal inflammation is characterised by significant elevations in PGE2, PGD2, TXB2, 5-HETE, 15-HETE, 12-HETE, and 11-HETE, with the profile of these lipid mediators correlating with histological severity, supporting the deployment of eicosanoid profiling as a quantitative tool for grading mucosal inflammation [24].

## 4. Lipid-Driven Pathogenic Pathways

### 4.1. Specialised Pro-Resolving Mediators and Resolution of Inflammation

Specialised pro-resolving mediators (SPMs) are endogenous bioactive lipids biosynthesised from omega-3 PUFAs through lipoxygenase-dependent enzymatic pathways. The principal SPM families, resolvins, protectins, and maresins, actively orchestrate resolution of inflammation by reprogramming immune cells, reducing cytokine production, enhancing clearance of cellular debris, and restoring tissue homeostasis without suppressing the immune response [22,23]. E-series resolvins (RvE) are derived from EPA, while D-series resolvins, maresins, and protectins arise from DHA; their receptor-mediated signalling operates through stereoselective activation of G protein-coupled receptors at nanomolar to picomolar concentrations [25]. RvE1, for example, activates ChemR23 while also serving as a partial agonist at the leukotriene B4 receptor BLT1, allowing simultaneous attenuation of pro-inflammatory signalling and promotion of resolution programmes [25].

Preclinical evidence supports a therapeutic role for SPMs in experimental colitis. Arita et al. showed that resolvin E1 protected mice from TNBS-induced colitis, with improvement in metabolic and histological parameters, reduction in TNF-α, IL-1β, IL-12, and NO, and limitation of leukocyte trafficking [22]. Bento et al. demonstrated that RvD2, AT-RvD1, and 17R-hydroxydocosahexaenoic acid improved the clinical profile and histopathological lesions of DSS- and TNBS-induced experimental colitis, reducing NF-κB expression and adhesion molecule levels [23]. Building on these findings, Chaim et al. showed that RvD2 and its omega-3 precursor reduce the disease activity index, colonic damage, and CXCL1 expression while increasing IL-10 in DSS-induced colitis in C57BL/6J mice, suggesting synergy with anti-TNF therapy and supporting RvD2 as a possible adjunct therapeutic in IBD [26].

Since omega-3 fatty acids are precursors of SPMs, their dietary deficiency may contribute to unresolved mucosal inflammation, providing a mechanistic rationale for the omega-3 supplementation strategies discussed in Section 6 [22,23,27,28].

### 4.2. Sphingolipid Dysregulation and Immune Homeostasis

Sphingolipid metabolism constitutes a critical node in the IBD pathogenic network. As the most reproducible class-level alteration noted in Section 3, it has both host-derived and microbiome-derived sphingolipids contributing to disease perpetuation or protection. Untargeted lipidomic analysis of UC mucosal biopsies revealed discriminant lipid signatures between treatment-naïve patients, deep-remission patients, and healthy controls, characterised by decreased phosphatidylcholine and ceramide species in inflamed mucosa [29].

Colonisation of mice with sphingolipid biosynthesis-deficient *Bacteroides fragilis* resulted in increased colonic invariant natural killer T cells, elevated IL-4 and IL-13 secretion, and exacerbated oxazolone-induced colitis compared to wild-type *B. fragilis* colonisation, demonstrating that commensal sphingolipids directly restrict pro-inflammatory ILC responses [30]. Across *Bacteroides* species, dihydroceramide phosphoethanolamine is the most abundant sphingolipid (constituting 19–29% of total bacterial lipids), and fluorescent labelling experiments confirmed its migration to the liver, colon, ileum, brain, and skin via intestinal epithelial transport, suggesting systemic participation in host-microbe sphingolipid homeostasis [31].

**Figure 1:**
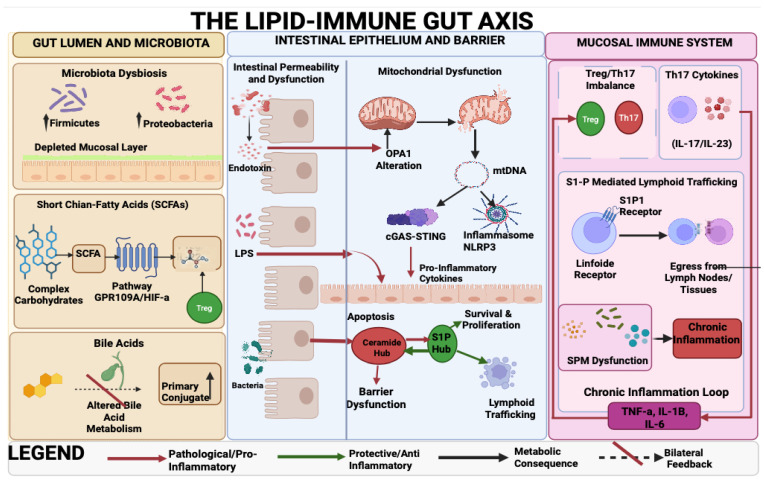
The lipid–immune gut axis in inflammatory bowel disease. The illustration summarises the three interconnected pathophysiological compartments through which lipid dysregulation contributes to persistent mucosal inflammation in IBD: the gut lumen and microbiota, the intestinal epithelial barrier, and the mucosal immune system. In the luminal compartment, microbial dysbiosis, characterised by depletion of beneficial Firmicutes and expansion of Proteobacteria, is associated with reduced short-chain fatty acid production, altered bile acid metabolism, and increased exposure to microbial products such as lipopolysaccharide. At the epithelial interface, impaired barrier integrity, increased intestinal permeability, mitochondrial dysfunction, inflammasome activation, apoptosis, and disruption of the ceramide/S1P balance amplify epithelial injury and pro-inflammatory signalling. Within the mucosal immune compartment, Th17/Treg imbalance, IL-17/IL-23-driven cytokine responses, S1P-mediated lymphocyte trafficking, and defective specialised pro-resolving mediator activity sustain immune-cell recruitment and chronic inflammation. Red arrows indicate pathological or pro-inflammatory pathways, green arrows protective or anti-inflammatory mechanisms, black arrows metabolic consequences or directional flow, and dashed arrows bidirectional feedback. Together, these processes create a self-reinforcing lipid–microbiota–barrier–immune loop that perpetuates intestinal inflammation in IBD.

At the host immune level, glucosylceramide, synthesised by UGCG, is essential for maintaining the Th17/Treg balance. In murine DSS colitis, UGCG knockdown reduced regulatory T cell populations and increased CD4 effector cells, while intravenous nanoparticle-delivered glucosylceramide improved colitis and restored splenic and colonic Treg populations [12]. Whether this axis is therapeutically tractable in human IBD is currently untested. Atypical intestinal sphingolipid metabolism also shapes gut microbiome composition by selectively increasing *Firmicutes* and *Verrucomicrobia* while promoting conjugated bile acid accumulation; in IBD patients, elevated colonocyte sphingosine kinase 2 (SphK2) has been associated with the development of metabolic dysfunction-associated steatotic liver disease, extending the consequences of intestinal sphingolipid dysregulation to systemic metabolic outcomes [32]. Figure 1 depicts the lipid immune gut axis and its alterations in IBD.

**Figure 2:**
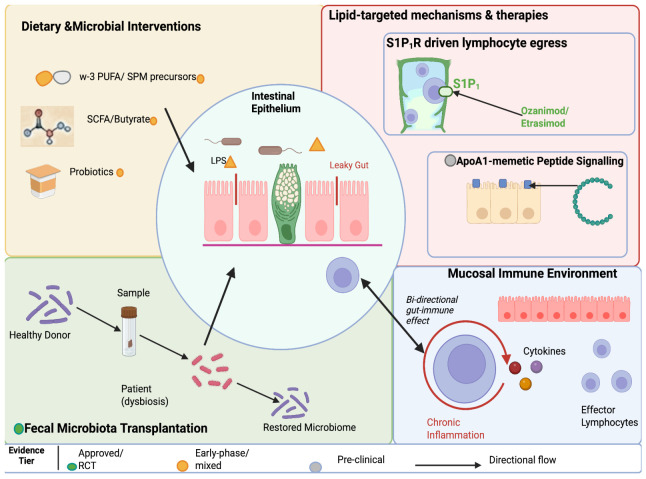
Lipid-targeted and microbiota-directed therapeutic strategies for restoring mucosal homeostasis in inflammatory bowel disease. The figure illustrates dietary and microbial interventions, including omega-3 PUFA/SPM precursors, SCFA/butyrate supplementation, probiotics, and faecal microbiota transplantation, alongside lipid-targeted mechanisms such as S1P receptor modulation and ApoA1-mimetic peptide signalling.

The main lipid-targeted and microbiota-directed therapeutic strategies that may contribute to mucosal immune restoration in IBD are summarised in Figure 2.

### 4.3. Phospholipid Remodelling and Barrier Dysfunction

Phospholipid metabolism is an active mediator of host-microbe crosstalk and barrier integrity [33]. Gut microbiome metabolism of dietary phosphatidylcholine generates lysophosphatidylcholine (LPC) and lysophosphatidic acid (LPA), which are elevated in the faeces of IBD patients and induce visceral hypersensitivity through TRPC5 and LPAR1/LPAR3-dependent mechanisms, directly connecting microbial lipid metabolism to the visceral pain experienced by IBD patients [34].Simultaneously, a suppressed one-carbon cycle in IBD patient tissues limits choline availability, impairing the interconversion of phospholipids and sphingolipids and producing a state of metabolic inflexibility characteristic of established disease [33].

Treatment-refractory IBD patients with adequate biological therapy trough levels but persistent non-response exhibit marked mucosal lipid shifts relative to treatment-naïve patients who subsequently achieved remission, including alterations in very-long-chain fatty acid metabolism (ELOVL3-related pathways) and distinct phospholipid species [29]. This intrinsic mucosal lipid dysregulation may contribute to biological therapy resistance independently of drug pharmacokinetics, identifying phospholipid profiles as possible predictive biomarkers for refractoriness.

### 4.4. The Bile Acid–Microbiome Axis

Bile acid metabolism exemplifies the bidirectional lipid-microbe relationship in IBD. IBD patients exhibit depletion of *Firmicutes bacteria* involved in bile acid biotransformation and enrichment of *Proteobacteria*, leading to impaired secondary bile acid production and accumulation of primary and conjugated bile acids [35]. The abundance of the bile acid-inducible (bai) operon in metagenomic samples is highly predictive of the secondary bile acid metabolic state, with bai genes from *Clostridium* species more prevalent in IBD patients than controls, thereby establishing specific bacterial genetic signatures as potential biomarkers for predicting metabolic dysregulation [36].

### 4.5. Immunometabolic Reprogramming

The immunometabolic landscape of IBD is characterised by the reprogramming of energy substrate utilisation across immune cell subsets. Effector T cells rely on TCR/mTOR-driven de novo lipogenesis to support proliferation and cytokine secretion, while regulatory T cells depend on fatty acid oxidation (FAO)-derived metabolites to sustain Foxp3 expression and suppressive function [37]. M1 macrophages utilise glycolysis and de novo lipogenesis, whereas M2 macrophages activate PPAR-γ and LXR pathways, promoting FAO and anti-inflammatory activity [37]. In IBD, the butyrate depletion described above (Section 3.6) compromises colonocyte energetics and Treg support, while triglyceride and phospholipid alterations are closely linked to pathogenesis and treatment response [37].

Cross-cohort integrative analysis of nine metagenomic and four metabolomics cohorts identified three organisms, *Asaccharobacter celatus*, *Gemmiger formicilis*, and *Erysipelatoclostridium ramosum*, with genes in two-component system pathways linked to faecal calprotectin [38].

## 5. Lipids as Emerging Biomarkers in IBD

Before surveying individual candidates, it is important to define the evidential lens applied throughout this section. We distinguish four tiers:

*discovery-stage* markers identified in a single, usually small, cross-sectional cohort without replication;
*replicated* markers showing a consistent direction of effect across two or more independent cohorts but without prospective or interventional testing;
*mechanistically grounded* candidates supported by model-system biology yet clinically unproven in humans;
*clinically actionable* markers that have been externally validated, benchmarked head-to-head against established tools such as CRP and faecal calprotectin, and shown to alter a management decision.

At present no lipidomic biomarker in IBD has reached this final tier, and the candidates below—cross-referenced to their validation status in Table 1 (Panel F)—sit predominantly at the discovery or replication stage. This distinction is maintained deliberately to avoid conflating biological plausibility with clinical readiness.

### 5.1. Diagnostic Biomarkers

The identification of blood or stool-based lipidomic signatures capable of accurately distinguishing IBD from non-IBD conditions has become a major investigative focus. The landmark contribution in this area is the study by Salihovic et al. (2024), which identified and validated a two-lipid blood-based diagnostic signature for paediatric IBD across three independent Scandinavian inception cohorts [9]. The signature comprised elevated lactosylceramide [LacCer] and reduced ether-linked phosphatidylcholine [PC], two species that are not broad markers of inflammation but appear specific to IBD. Across all three cohorts, this two-analyte signature outperformed high-sensitivity CRP and achieved diagnostic performance comparable to faecal calprotectin without requiring stool collection. LacCer is recognised by innate immune receptors including natural killer T cells and toll-like receptors, and its elevation reflects innate immune activation concurrent with disrupted glycosphingolipid catabolism at the inflamed intestinal epithelium. Conversely, depletion of ether-linked PC reflects impaired peroxisomal biogenesis and oxidative phospholipid metabolism [9]. The multi-cohort inception design employed by Salihovic et al. is the methodological reference point for this field, and is examined as such in Section 7.4 [9].

Tews et al. (2024) performed 1H-NMR metabolomic and lipidomic profiling in the largest NMR-based IBD cohort to date (CD n = 55, UC n = 34, healthy controls n = 40), achieving cross-validated AUROC values exceeding 0.90 for IBD versus healthy controls [20]. Key discriminating features included perturbations in VLDL and LDL subclass triglyceride and phospholipid fractions, and decreased ApoA1 and ApoA2. This study demonstrated that IBD-associated lipidomic perturbations carry dual utility as both diagnostic and cardiovascular risk biomarkers [20].

Guan et al. (2020), applying UPLC-QTOF-MS untargeted plasma lipidomics, identified 55 significantly altered metabolites in IBD versus healthy controls across five lipid classes (fatty acyls, glycerophospholipids, prenol lipids, sphingolipids, and sterol lipids) achieving sensitivity and specificity above 80% in an independent validation cohort [39]. Across these three diagnostic studies the evidential weight is uneven as only Salihovic et al. provides genuine external validation across independent inception cohorts, whereas the metrics of Tews et al. rest on internal cross-validation and those of Guan et al. on a single validation set. Their reported accuracies should therefore be read as replication and discovery-grade signals of feasibility rather than as established diagnostic performance.

### 5.2. Lipidomic Differentiation Between CD and UC

Accurate sub-classification of IBD into CD and UC carries fundamental therapeutic consequences, as approximately 10% of IBD cases cannot be definitively classified at diagnosis. A methodologically rigorous study that focused on CD-specific characterisation was reported by Ferru-Clément et al. (2023), who applied untargeted high-resolution LC-QTOF-MS covering 25 lipid subclasses to serum from 600 CD patients and 600 matched controls across two independent cohorts [40]. Over 70 lipid features were strongly associated with CD, and classification models achieved AUROC values from 0.84 to 0.97 using as few as 5–9 lipid species. The most discriminating features reflected mechanistically distinct pathological processes: a phosphatidylethanolamine ether species indicating peroxisomal plasmalogen dysfunction; a sphingomyelin bearing an odd-chain fatty acid reflecting reduced microbial propionate availability; a cholesterol ester reflecting systemic lipid transport dysfunction; a very-long-chain dicarboxylic acid reflecting peroxisomal β-oxidation capacity; and sitosterol sulphate reflecting intestinal sterol absorption impairment [40].

Tews et al. (2024) provided clinically actionable inter-subtype discrimination through NMR-based lipoprotein subclass analysis, demonstrating significantly elevated VLDL-5 triglycerides and phospholipids in CD relative to UC, and significantly reduced LDL-2 phospholipids, ApoB, and LDL-2 particle number in CD compared to UC, thereby reflecting the more extensive metabolic disruption in CD secondary to small bowel involvement and fat malabsorption [20]. The persistence of elevated phosphatidylserine in CD patients both during active disease and remission, as demonstrated by Iwatani et al. (2020), represents a disease-state-independent CD-specific marker not observed at equivalent levels in UC, and may reflect ongoing subclinical immune cell turnover in the mucosa [41]. They profiled 698 molecular species across 22 lipid classes and demonstrated that lysophosphatidic acid, lysophosphatidylserine and S1P are elevated in UC, while LPC and PC are decreased in CD, thus providing both shared and subtype-specific diagnostic features [41]. Multi-sample profiling across serum, faeces, and colonic mucosa showed that UC in remission with IBS-like symptoms is lipidomically distinct from diarrhoea-predominant IBS [42]. Lipidomic signatures therefore carry biological specificity beyond clinical phenotype, even when symptom burdens overlap.

Studies supporting lipidomic differentiation between CD and UC are summarised in Table 2. 

### 5.3. Prediction of Onset, Disease Activity & Relapse

Beyond cross-sectional diagnosis, lipidomic biomarkers have demonstrated capacity to track mucosal inflammation quantitatively, predict relapse in quiescent disease, and serve as pharmacodynamic markers of therapeutic response.

Using pre-diagnostic profiling of prospectively collected biobank samples, Hua et al. (2023) showed that IBD-associated lipid-pathway disruptions are measurable in blood several years before clinical diagnosis [43]. This places lipid dysregulation within the pre-diagnostic pathogenic cascade and suggests a window for early interception through lipidomic screening in high-risk populations [43].

Mendelian randomisation by Long et al. (2024) [44] linked genetically determined sterol esters, phosphatidylcholines, sphingomyelins, and phosphatidylethanolamines to IBD risk, with the associations mediated by circulating inflammatory proteins such as CD6 and CCL4. Notably, elevated sphingomyelin was causally protective against UC [44]. A complementary 2025 Mendelian randomisation study identified PD-L1 as a mediator of plasma lipid effects on UC risk, suggesting that lipid-mediated immune checkpoint regulation represents a novel mechanism linking metabolic and immune dysregulation [45]. Yin et al. (2025) further confirmed statistically significant causal effects of lysophosphatidylcholine and phosphatidylcholine on UC risk through inverse-variance weighted analysis [46].

Tews et al. (2025) reported a significant decline in serum LPC species specifically in patients with severe IBD, reflecting heightened phospholipase A2 (PLA2) activity rerouting membrane phospholipids toward eicosanoid synthesis, thereby creating a pharmacodynamic lipid fingerprint of severe mucosal inflammation that may function as a continuous severity scale from remission to severe active disease [47].

Yang et al. (2022) [48] profiled plasma lipidomics in CD patients before and after exclusive enteral nutrition (EEN), identifying seven lipid classes altered in active CD, four of which, PC, PS, phosphatidylinositol (PI), and simple glucosylceramide, normalised to control levels following clinical remission. Pre-treatment lipid class levels correlated significantly with the Crohn’s Disease Activity Index (CDAI) and systemic inflammatory markers including hs-CRP [48].

Bjerrum et al. (2022) demonstrated through serial colonic biopsy lipidomics that distinct phospholipid and oxylipin trajectories characterise delayed mucosal wound healing even in clinically and endoscopically quiescent UC, identifying a subset of patients at elevated relapse risk not captured by colonoscopy alone [49]. This is relevant given the known dissociation between clinical, endoscopic, and histological remission in UC, and the unpredictable nature of the disease course [49].

### 5.4. Prognostic Biomarkers and Therapy Response Prediction

The clinical imperative for pre-treatment therapy response biomarkers in IBD is quantified by PANTS cohort data: Chanchlani et al. (2024), reporting three-year outcomes from 1610 anti-TNF-naïve CD patients initiating infliximab or adalimumab across 92 UK sites, found that only 43.0% achieved remission at week 54 and that loss of response occurred at 19.5% per year in initial responders [50]. These prospective data frame the therapeutic window that lipidomic predictive biomarkers could address.

The proof-of-concept lipidomic investigation of biological therapy response is the pilot study by Rioux et al. (2024), which performed serum lipidomics across over 1100 lipid entities in 92 IBD patients at baseline and 14 weeks after initiating vedolizumab, identifying baseline lipidomic features differentiating subsequent responders from non-responders and on-treatment lipid changes most pronounced in clinical responders [51].

Ferru-Clément et al. (2023) demonstrated that CD-specific lipid classifiers are differentially associated with disease behaviour, specifically the B2 (stricturing) and B3 (penetrating) phenotypes of the Montreal classification, independently of disease location [40]. Sphingomyelins bearing very-long-chain fatty acids were specifically depleted in complicated disease behaviour, implicating impaired ceramide synthase 2 (CerS2) activity or peroxisomal dysfunction as drivers of CD complications, and raising the prospect of lipidomic-guided early escalation to advanced therapies [40].

Diab et al. (2025) demonstrated that treatment-refractory UC patients show marked mucosal lipid shifts compared to all control groups, with significant decreases in ceramides, sphingomyelins, PC, and PE compared to treatment-naïve patients; while IBD patients in biological remission showed mucosal lipid profiles comparable to healthy controls, establishing remission as a state of mucosal lipid normalisation and nominating mucosal lipidomics as a potential pre-treatment predictor of biological refractoriness [29].

## 6. Future Translational Perspectives

The mechanistic insights emerging from lipidomic research in IBD suggest several translational directions, though further investigation is needed before therapeutic application. However, the strategies below are pending interventional human evidence in IBD: the supporting data are genetic, preclinical, or observational, and these interventions remain hypotheses for testing which in time could become approaches for clinical deployment.

### 6.1. Lipoprotein-Directed Strategies

Genetic and experimental evidence implicates lipoprotein metabolism as a modifiable axis in IBD pathogenesis. CETP inhibition with evacetrapib attenuated experimental colitis in a murine model through HDL-C elevation, without affecting LDL-C or triglycerides [52,53], and Mendelian randomisation data suggest that CETP suppression may reduce CD risk, whereas PCSK9 inhibition may paradoxically increase IBD susceptibility implying that the direction and mechanism of lipoprotein modulation matter considerably [19]. Retrospective clinical data have also associated statin use with reduced IBD onset risk [27]. Whether these observations translate into viable therapeutic strategies will depend on prospective trials in IBD populations, which have not yet been undertaken.

### 6.2. ApoA1 Mimetic Peptides

Short ApoA1 mimetic peptides (4F, Tg6F) restate the anti-inflammatory functions of HDL-associated apolipoprotein A-I and have shown efficacy in the Cox2-MKO/CCHF model of CD-like ileocolitis, reducing intestinal inflammation and improving histopathological indices even when administered after disease establishment [53]. These findings provide proof-of-concept that HDL-mimetic approaches can target intestinal lipid peroxidation and inflammatory signalling, but translation to human IBD remains distant, as no clinical trials have been initiated and the pharmacokinetics of oral peptide delivery require optimisation.

### 6.3. Short-Chain Fatty Acid Supplementation

SCFA supplementation represents perhaps the most clinically advanced lipid-based intervention in IBD. Meta-analytic data suggest that SCFA enemas may contribute to remission in UC, potentially synergising with 5-ASA and oral supplementation over 6–12 weeks has been associated with improved inflammatory biomarkers in active UC [28,54]. However, the independent therapeutic contribution of SCFAs has not been isolated from combination regimens, and optimal formulation, dosing, and patient selection criteria remain undefined. These are critical gaps that future randomised controlled trials will need to address.

### 6.4. Sphingolipid-Axis Modulation

The sphingolipid axis demonstrates that lipid-signalling pathways are inducible in IBD. Ozanimod, a selective sphingosine-1-phosphate (S1P) receptor 1 and 5 modulator, achieved induction and maintenance efficacy in moderately-to-severely active ulcerative colitis in the phase 3 True North trial and is now approved for this indication [55]. Mechanistically, it acts by internalising S1P_1_ receptors on lymphocytes and restricting their egress from lymphoid tissue into inflamed mucosa, rather than by correcting a measured lipidomic abnormality [55].

It is therefore important to frame ozanimod accurately: it was developed through lymphocyte-trafficking immunology, not through lipidomic biomarker discovery. Nonetheless, its success is conceptually significant for this review, as it confirms that a node of the sphingolipid rheostat repeatedly implicated in IBD lipidomic studies is not merely a correlative marker but a tractable point of intervention. Whether upstream elements of the same axis, such as the glucosylceramide-dependent regulation of mucosal tolerance discussed above [31], can be exploited therapeutically in humans remains untested, but the clinical arrival of an S1P-directed agent provides a strong rationale for pursuing them.

The current evidence supporting proposed lipid-based therapeutic strategies in IBD, ranging from preclinical models to human interventional data, is summarized in Table 3.

## 7. Challenges and Reproducibility in IBD Lipidomics

Despite the compelling accumulation of lipidomic evidence in IBD, significant methodological, biological, and translational barriers currently prevent the deployment of lipid biomarker panels in routine clinical practice. Table 4 summarizes the key methodological limitations currently constraining reproducibility and clinical translation in IBD lipidomics, together with practical recommendations for their mitigation.

### 7.1. Analytical Variability and Inter-Platform Reproducibility

The reproducibility of lipidomic identifications across analytical platforms is a fundamental and insufficiently appreciated challenge. Von Gerichten et al. (2024) quantified this directly in a head-to-head comparison of two widely used software platforms, MS-DIAL (v4.9.221218) and Lipostar (v2.1.4) (Table 2): identical LC-MS data yielded poor inter-platform agreement in lipid identifications, improved only modestly by tandem MS confirmation [56]. The root causes are algorithmic: differences in spectral processing, database coverage and annotation stringency, adduct modelling, and peak alignment meaning that two laboratories can derive divergent identifications from the same raw spectra. For closely related species such as regioisomers and double-bond positional isomers, particularly prevalent among glycerophospholipids and ether lipids, these algorithmic differences generate fundamentally divergent identifications from equivalent raw data.

### 7.2. Pre-Analytical Variability and Sample Matrix Effects

The quantitative lipidome is exquisitely sensitive to pre-analytical conditions. Reis et al. (2021) demonstrated that storage even at 4 °C produces progressive lipid concentration changes within 3 days, while benchtop or heated storage rapidly amplifies degradation of free fatty acids, lysophospholipids, and eicosanoids, the precise lipid classes most frequently reported as IBD biomarkers [57]. A single freeze–thaw cycle produces measurable effects on LPC and diacylglycerol concentrations; comparisons between studies using serum versus EDTA-plasma are further complicated by LPC and diacylglycerol elevation from platelet activation during serum clotting [57]. Drug effects represent an additional source of pre-analytical noise: Li et al. (2023) demonstrated that thiopurine-induced leukopenia is associated with a distinct plasma lipidomic perturbation profile, directly attributable to drug-induced leukocyte depletion rather than disease activity, thus providing direct evidence that concomitant immunomodulator use can generate false-positive disease associations in biomarker discovery analyses [59].

### 7.3. Isobaric Overlap and Quantification Challenges

Intrinsic mass spectrometric challenges substantially limit confident lipid identification and quantification. Höring et al. (2021) systematically demonstrated that co-occurrence of multiple lipid species at identical or near-identical *m*/*z* values introduces systematic quantification errors of clinically relevant magnitude across glycerophospholipid and sphingolipid classes without dedicated isotope correction algorithms [58]. Adduct formation variability, whereby individual lipid species generate multiple ionic adducts ([M+H]^+^, [M+Na]^+^, [M+NH_4_]^+^) with relative proportions varying by up to 70% depending on molecular structure and ionisation conditions, exacerbates quantification inaccuracy in the absence of class-specific isotope-labelled internal standards [58].

### 7.4. Small Cohort Sizes and Absence of Independent Validation

The majority of published IBD lipidomic studies report discovery findings from cohorts of 20–200 patients without independent external validation in geographically or ethnically distinct populations. Small discovery cohorts are inherently susceptible to overfitting: untargeted lipidomic workflows generate thousands of candidate features tested against limited sample sizes, producing discriminant statistics that reflect sample-specific noise rather than generalisable disease biology. The study by Salihovic et al. (2024), having validated its two-lipid signature across three independent inception cohorts with consistent performance; by contrast, the 20–50 patient cohorts typical of most published IBD lipidomic studies cannot support complex statistical models that capture the heterogeneous IBD spectrum, including the effects of disease location, duration, prior therapy, and concomitant medications, all of which interact with the circulating lipidome in disease-specific ways [9,59].

### 7.5. Confounding by Diet, BMI, and Biological Variables

Plasma fatty acid and phospholipid profiles are directly influenced by dietary fat composition, the omega-3/omega-6 ratio, and total caloric intake. IBD patients frequently adopt therapeutic exclusion diets or enteral nutrition interventions that alter habitual lipid intake: a confound demonstrated directly by Yang et al. (2022), who observed normalisation of four plasma lipid classes after EEN induction [48]. Geographic and ethnic variation in dietary patterns may substantially contribute to inter-cohort lipidomic heterogeneity. Tews et al. (2024) further confirmed that sex, age, and BMI exert significant effects on lipoprotein subclass compositions and apolipoprotein levels in IBD cohorts, confirming that these biological variables must be systematically covaried in any multivariate lipidomic model intended for clinical translation [20].

Because habitual diet is among the strongest determinants of the circulating and faecal lipidome, dietary status should be treated as a measured covariate rather than background noise. Future IBD lipidomic studies should, at minimum, capture habitual intake with a validated instrument. This can be achieved by a food frequency questionnaire or multi-day food diary. Quantifying the omega-6/omega-3 ratio while also recording all exclusion diets, enteral or parenteral nutrition, and lipid-relevant supplements (fish oil, fibre, probiotics); standardise and document fasting duration and sampling time to limit diurnal and post-prandial variation; and enter dietary descriptors as explicit covariates in multivariable models. Where feasible, a short standardised-diet run-in before sampling might further reduce diet-driven between-subject variance and improve the interpretability of disease-associated signals.

### 7.6. Lack of Longitudinal, Endoscopy-Integrated Study Designs

The vast majority of published IBD lipidomic studies are cross-sectional in design, capturing a single lipid snapshot at one disease state without temporal tracking across disease phases. Clinical-grade IBD biomarker validation requires demonstration that a candidate marker correlates prospectively with endoscopically assessed mucosal inflammation, precedes clinical relapse, and changes with therapeutic intervention, a standard met by only a small fraction of existing studies. Future IBD lipidomic investigations must be designed prospectively, with multiple sampling time points aligned to pre-specified endoscopic outcomes, across geographically diverse multicentre cohorts, and with rigorous pre-analytical standardisation. Only through such designs can lipidomic biomarkers achieve the clinical-grade evidence required for regulatory approval and routine implementation in IBD management.

### 7.7. Minimum Validation Criteria for Clinically Oriented Lipidomic Biomarkers

The barriers above can be reframed constructively as a checklist that a candidate lipid biomarker should satisfy before it is proposed for clinical use. We suggest the following minimum criteria:

   (i) independent external validation in at least one cohort distinct from the discovery population, ideally of differing geography and ethnicity
  (ii) standardised, pre-registered sample handling with full pre-analytical reporting (tube type, processing time, storage temperature, freeze–thaw count)
 (iii) explicit correction for diet, body mass index, medication, and disease activity
 (iv) a predefined, locked lipid panel and analytic pipeline rather than post hoc feature selection
   (v) demonstrated reproducibility across platforms and laboratories, using class-specific isotope-labelled internal standards and MS/MS-level confirmation
  (vi) head-to-head comparison against established biomarkers such as CRP and faecal calprotectin
 (vii) clinically meaningful, prospectively defined cut-offs rather than cohort-optimised thresholds
(viii) evidence that applying the biomarker changes a clinical decision or outcome. Criteria (i)–(v) establish analytical and statistical credibility, whereas (vi)–(viii) establish clinical value. A marker meeting only the former remains a discovery tool, and only one satisfying the latter approaches actionable status. Against this framework, the paediatric signature of Salihovic et al. currently advances furthest, while most reported candidates satisfy few of these conditions.

## 8. Discussion

This review supports the concept that lipidomics may contribute to a broader transition in IBD from conventional clinical classification toward biochemical disease characterisation. Current disease labels, including CD, UC, IBD-unclassified, disease location, behaviour, and activity scores, remain essential for clinical communication and treatment decisions. However, they only partially capture the biological heterogeneity that drives disease onset, progression, complications, and response to therapy. The evidence summarised in this review indicates that lipidomic signatures may reflect several biologically relevant dimensions of IBD, including epithelial barrier disruption, immune activation, failed resolution of inflammation, microbiome-derived metabolic dysfunction, bile acid perturbation, and systemic lipoprotein remodelling. This biochemical reclassification could be particularly relevant across distinct disease phenotypes. In early disease, lipidomic perturbations detected before or around diagnosis may help identify patients with an active preclinical inflammatory trajectory, opening a potential window for early interception and risk-stratified monitoring. In late or long-standing disease, persistent lipid abnormalities may reflect cumulative tissue remodelling, chronic epithelial injury, metabolic inflexibility, or incomplete resolution despite apparent clinical control. In perianal disease, where current biomarkers often fail to adequately represent local tissue inflammation and fistula biology, lipidomic profiling may offer a complementary approach to characterise immune-metabolic pathways involved in penetrating complications, although dedicated studies in this phenotype are still lacking. In difficult-to-treat or treatment-refractory disease, mucosal and circulating lipidomic signatures may help distinguish patients with pharmacokinetic failure from those with intrinsic biological refractoriness, thereby supporting more rational therapeutic sequencing. Similarly, complicated Crohn’s disease phenotypes, including stricturing and penetrating behaviour, may be better understood through lipidomic markers of sphingolipid, phospholipid, and peroxisomal dysfunction rather than through morphology alone.

The findings reviewed here are consistent with this vision. Diagnostic studies have identified lipidomic signatures capable of discriminating IBD from healthy controls and, in some cases, differentiating CD from UC. Other studies suggest that lipidomic alterations correlate with disease activity, endoscopic severity, mucosal healing, relapse risk, and response to therapy. Particularly relevant is the observation that lipid pathway disruptions may be detectable before clinical diagnosis, suggesting that metabolic remodelling is not merely a consequence of established inflammation but may participate in the earliest phases of disease development. Likewise, the association between specific lipid profiles and treatment-refractory disease supports the hypothesis that lipidomics may help identify disease biology that is not captured by CRP, faecal calprotectin, endoscopy, or traditional clinical indices.

Several limitations still prevent lipidomics from modifying routine IBD classification and the methodological constraints detailed earlier (Section 7; Table 2) apply with equal force to any reclassification scheme: most studies remain cross-sectional, single-centre, and modestly powered, lipidomic profiles are strongly shaped by diet and other biological and analytical covariates that are often incompletely controlled, and few candidate markers have been validated against hard clinical endpoints. The specific requirement for an endotype framework is more stringent as it must be shown that lipid-defined subgroups are reproducible across platforms and populations and that they predict disease course or treatment response independently of, and ideally better than, conventional classification.

Establishing this will require prospective, multi-cohort studies spanning the disease phenotypes above, with serial sampling aligned to the clinical, endoscopic, dietary, and microbiome measures already outlined as future priorities (Section 7). Lipidomic models also need to be tested head-to-head against existing biomarkers. Critically, reclassification is unlikely to emerge from lipidomics in isolation: biologically coherent subtypes such as failed pro-resolving mediator production, sphingolipid-driven immune activation, bile acid–microbiome disruption, phospholipid-mediated barrier dysfunction, or systemic lipoprotein remodelling will be defined only by integrating lipidomic data with transcriptomic, proteomic, metagenomic, and immunophenotypic profiles. Such molecularly defined subgroups may ultimately prove more informative than conventional labels in predicting disease course and guiding treatment selection.

## 9. Conclusions

Lipidomics offers a promising route toward a biochemical characterisation of IBD that could complement and eventually reshape traditional classification systems. At present, its greatest value lies in revealing disease mechanisms and generating candidate biomarkers for diagnosis, prognosis, and therapeutic stratification. Its future clinical impact will depend on rigorous standardisation, multicentre validation, longitudinal study designs, and integration with other omics and clinical datasets. If these challenges are addressed, lipidomics may contribute to a new taxonomy of IBD in which patients are classified not only by disease location, behaviour, and symptoms, but by the molecular pathways that drive their individual disease trajectory.

## Figures and Tables

**Table 1:** The clinical pipeline of lipidomics in IBD: from biological sample to clinical decision-making. This multi-panel table maps the translational pipeline from sample collection through analytical interrogation, lipid class identification, clinical application, and barriers to implementation. Panel A: Sample Types. Panel B: Analytical Platforms. Panel C: Lipid Classes Detected. Panel D: Clinical Applications. Panel E: Barriers to Clinical Implementation. Panel F: Key Lipid Biomarker Candidates. Panel F summarises key biomarker candidates with their current validation status.

**Panel A**				
**Sample Type**	**Collection Method**	**Advantages**	**Limitations**	**IBD Evidence Level**
Serum/Plasma	Venipuncture	Minimally invasive, scalable; reflects systemic lipid milieu	Does not capture mucosal-specific changes; serum ≠ EDTA-plasma	Most studied matrix in IBD lipidomics
Mucosal Biopsy	Endoscopic biopsy; paired inflamed vs. non-inflamed	Site-specific tissue lipidome	Invasive; requires endoscopy; small tissue mass	Gold standard; enables MALDI-MSI spatial lipidomics
Stool	Non-invasive; home sampling feasible	Host + microbial lipid signatures; integrates luminal environment	Pre-analytical variability (water content, transit time)	Emerging paediatric utility; faecal LacCer as diagnostic lipid
Exhaled Breath/Urine	Exhaled breath condensate; spot urine	Non-invasive; point-of-care potential	Very limited lipid coverage	Limited IBD data; exploratory only
**Panel B**				
**Platform**	**Methodology**	**Strengths**	**Limitations**	**IBD Relevance**
LC-MS/MS	Targeted & untargeted; RPLC/HILIC separation; MRM or DDA/DIA	High sensitivity & specificity; broad lipid coverage	Software-dependent ID variability (14% cross-platform agreement)	Workhorse for clinical IBD lipidomics
GC-MS	FA profiling after derivatisation; volatile metabolite analysis	High reproducibility	Limited to volatile lipids; no intact sphingolipids	Robust for faecal SCFA profiling
MALDI-MSI	Spatial lipidomics on tissue sections; laser desorption imaging	In situ lipid mapping; cellular-level spatial resolution	Requires biopsy; limited class coverage	Resolves inflamed vs. adjacent mucosa
Shotgun/DIMS	Direct infusion; no chromatographic separation	Rapid screening; high throughput	Lower resolution; isobaric overlap	Rapid screening; lower resolution than LC-MS
NMR Spectroscopy	Nuclear magnetic resonance	Non-destructive; highly reproducible	Limited lipid coverage; low sensitivity vs. MS	Complementary to MS approaches
**Panel C**				
**Lipid Class**	**Key Species**	**Biological Role in IBD**	**Primary Platform(s)**	
Sphingolipids	Ceramide, S1P, SM, GluCer, LacCer	Central to ceramide/S1P rheostat; strongest IBD signal; barrier	LC-MS/MS; MALDI-MSI	
Glycerophospholipids	PC, PE, LPC, LPE, PI, PS	Membrane remodelling markers; LPC depletion tracks inflammation and predicts therapy non-response	LC-MS/MS; Shotgun/DIMS	
Eicosanoids/SPMs	PGE_2_, LTB_4_, LXA_4_, RvD1, RvE1	Pro-resolving vs. pro-inflammatory balance	LC-MS/MS (targeted); GC-MS	
Fatty Acids	SCFA (butyrate), ω-3/ω-6 PUFA, oxylipins	Microbiome–host interface; butyrate depletion reflects dysbiosis;	GC-MS (SCFA); LC-MS/MS (oxylipins)	
Bile Acids	Primary BAs; Secondary (DCA, LCA)	FXR/TGR5 axis; microbiome-dependent; conjugation patterns diverge in CD vs. UC	LC-MS/MS; GC-MS	
Sterols/Oxysterols	Cholesterol, 25-HC, 27-HC	Immune modulation via LXR signalling; oxysterol accumulation in inflamed mucosa	LC-MS/MS; GC-MS	
**Panel D**				
**Clinical Application**	**Candidate Biomarker(s)**	**Key Evidence**	**Current Limitations**	
IBD Diagnosis	LacCer(d18:1/16:0); PC(18:0/p22:6)	LacCer: top faecal diagnostic lipid (paediatric); PC(18:0/p22:6): IBD vs. healthy (AUC 0.85–0.95)	Single-centre discovery; no head-to-head vs. calprotectin	
CD vs. UC Differentiation	Ceramide chain-length profiles; PGE_2_/LXA_4_ ratio; bile acid conjugation	Distinct ceramide acyl-chain distributions; eicosanoid ratios diverge; bile acid patterns differ	Overlapping profiles in indeterminate colitis; medication confounding	
Disease Activity	LC/VLC ceramide ratio; LPC; faecal SCFA	LC/VLC ratio ↑ with endoscopic severity; LPC ↓ tracks inflammation; butyrate depletion reflects dysbiosis	Ceramide ratio in 2–3 cohorts, not prospectively validated; butyrate non-specific	
Relapse Prediction	SPMs (RvD1, RvE1, LXA_4_); S1P	SPM deficiency predicts failed resolution; persistent S1P ↑ in remission signals subclinical activity	Longitudinal sampling required; clinical data sparse; no cut-offs	
Therapy Response	Baseline sST2/ceramide; serum LPC	sST2/ceramide may guide biologic selection; LPC ↓ predicts non-response	Preliminary and retrospective; no RCT-embedded validation	
**Panel E**				
**Barrier**	**Specific Challenges**	**Impact**	**Proposed Solutions**	
Pre-Analytical Variability	Collection handling, freeze–thaw, fasting status, diurnal variation; no IBD lipidomics SOP	Batch artefacts indistinguishable from biological signal	Mandatory pre-analytical reporting (temperature, time, tube type, freeze–thaw count)	
Analytical Standardisation	Cross-platform reproducibility limited	14% ID agreement between platforms; adduct variability up to 70%	Class-specific isotope-labelled IS; mandate software/database reporting; MS/MS confirmation	
Cohort & Study Design	Small cohorts (n < 50); no multi-centre validation; diet/BMI/medication confounders	Overfitting; poor generalisability; inflated effect sizes	External validation in distinct cohorts; biological covariates; prospective serial sampling	
Bioinformatic Integration	Multi-omics fusion (lipidome + proteome + microbiome + transcriptome) immature	Siloed analyses miss cross-omic disease drivers	Integrated pipelines with standardised formats and shared ontologies	
Clinical Translation Gap	No FDA/EMA-approved lipid biomarker; cost vs. CRP/calprotectin unproven; no CLIA infrastructure	No regulatory pathway; reimbursement unlikely without comparative data	Embed panels in RCTs; head-to-head vs. existing biomarkers; point-of-care assays	
**Panel F**				
**Lipid/Ratio**	**Sample**	**Application**	**Key Finding**	**Validation Status**
LacCer(d18:1/16:0)	Stool	IBD diagnosis (paediatric)	Top discriminatory faecal lipid	Single-centre; needs validation
LC/VLC ceramide ratio	Serum/Biopsy	Disease activity	Ratio ↑ correlates with endoscopic severity	Replicated in 2–3 cohorts
LPC (multiple) ↓	Serum	Therapy response	LPC ↓ predicts biologic non-response	Preliminary; retrospective
PC(18:0/p22:6)	Serum	IBD diagnosis	IBD vs. healthy; AUC > 0.90	Discovery phase
RvD1/RvE1/LXA_4_	Biopsy/Serum	Relapse/chronicity	SPM deficiency → failed resolution	Mechanistic strong; clinical sparse
Faecal SCFA (butyrate ↓)	Stool	Dysbiosis/activity	Butyrate depletion correlates with inflammation	Well-replicated

Abbreviations: LC-MS/MS, liquid chromatography–tandem mass spectrometry; GC-MS, gas chromatography–mass spectrometry; MALDI-MSI, matrix-assisted laser desorption/ionisation mass spectrometry imaging; DIMS, direct infusion mass spectrometry; NMR, nuclear magnetic resonance; SCFA, short-chain fatty acid; PUFA, polyunsaturated fatty acid; SPM, specialised pro-resolving mediator; SM, sphingomyelin; S1P, sphingosine-1-phosphate; LPC, lysophosphatidylcholine; BA, bile acid; IS, internal standard; SOP, standard operating procedure; RCT, randomised controlled trial. The arrows indicate changes relative to the reference value, with upward arrows denoting increased values and downward arrows denoting decreased values.

**Table 2:** Lipidomic differentiation between Crohn’s disease and ulcerative colitis: study design, discriminating lipid features, comparative patterns, and mechanistic interpretation. The arrows indicate changes relative to the reference value, with upward arrows denoting increased values and downward arrows denoting decreased values.

Study (Year) [Ref]	Approach & Cohort	Discriminating Lipid Feature(s)	Pattern & Comparison Performed	Mechanistic Interpretation
Ferru-Clément et al., 2023 [40]	Untargeted LC-QTOF-MS, 25 lipid subclasses; serum, 600 CD vs. 600 matched controls across two independent cohorts (externally validated)	PE ether (plasmalogen) species; odd-chain sphingomyelin; cholesterol ester; very-long-chain dicarboxylic acid; sitosterol sulphate	CD-specific classifier; 5–9 species identify CD at AUROC 0.84–0.97. Comparison: CD vs. controls, not a direct CD–UC contrast	Peroxisomal plasmalogen dysfunction; reduced microbial propionate; systemic lipid-transport dysfunction; impaired peroxisomal β-oxidation; intestinal sterol malabsorption
Tews et al., 2024 [20]	^1^H-NMR lipoprotein subclass analysis; serum; single cohort	VLDL-5 triglycerides & phospholipids; LDL-2 phospholipids; ApoB; LDL-2 particle number	↑ VLDL-5 triglycerides and phospholipids in CD vs. UC; ↓ LDL-2 phospholipids, ApoB, and LDL-2 particle number in CD vs. UC. Comparison: direct CD vs. UC	Greater metabolic disruption in CD from small-bowel involvement and fat malabsorption
Iwatani et al., 2020 [41]	MS-based profiling, 698 species across 22 lipid classes; plasma; single cohort (discovery)	Phosphatidylserine (PS); lysophosphatidic acid, lysophosphatidylserine, S1P; LPC, PC	PS persistently elevated in CD across active disease and remission (proposed state-independent CD marker); LPA, lysophosphatidylserine, S1P elevated in UC; LPC and PC decreased in CD. Comparison: CD vs. UC vs. controls	PS proposed to reflect ongoing subclinical mucosal immune-cell turnover in CD; yields shared and subtype-specific features

**Table 3:** Evidence tiers for proposed lipid-based therapeutic strategies in IBD. Dashes denote no direct evidence identified; “clinical readiness” reflects the highest tier reached in human IBD.

Strategy (Target)	Preclinical	Observational Human	Interventional Human	Clinical Readiness
CETP inhibition/HDL-C elevation (evacetrapib)	Attenuates murine colitis via HDL-C rise [53]	MR: lower CETP linked to reduced CD risk [19]	None	Hypothesis only; no IBD trials
PCSK9 modulation	—	MR: PCSK9 inhibition linked to increased IBD susceptibility [19]	None	Not a viable target; possible harm signal
Statins (HMG-CoA reductase)	Indirect anti-inflammatory rationale	Retrospective: statin use linked to lower IBD onset [52]	None	Low; confounding-prone signal
ApoA1 mimetic peptides (4F, Tg6F)	Reduced ileocolitis in Cox2-MKO/CCHF model, effective post-onset [28]	—	None initiated	Very low; oral peptide PK unresolved
Glucosylceramide repletion (sphingolipid axis)	Nanoparticle glucosylceramide restores Tregs, improves murine colitis [31]	—	None	Very low; murine proof-of-concept only
S1P-receptor modulation (ozanimod)	S1P_1_/S1P_5_ modulation sequesters lymphocytes from inflamed mucosa [55]	—	Phase 3 True North RCT: induction + maintenance efficacy in moderate–severe UC [55]	Approved (FDA/EMA 2021) for moderate–severe UC
Omega-3/SPM precursors	Resolvins (RvE1, RvD1/D2) protect in murine colitis [22,23]	—	RCT/meta-analysis inconclusive; no robust CD maintenance benefit [6]	Low; not supported for maintenance
SCFA/butyrate supplementation	Colonocyte energetics, Treg support	—	Small enema/oral trials + meta-analysis: possible UC remission, 5-ASA synergy; effect not isolated [25,54]	Most advanced of the discovery-stage options; unproven independently

**Table 4:** Overview of the key methodological limitations reported in lipidomics studies and corresponding recommendations for their mitigation.

Reference	Study Design	Main Finding & Limitation	Recommendation
Von Gerichten et al., 2024 [56]	Multi-platform LC-MS software comparison	Only 14% lipid ID agreement between platforms; MS/MS confirmation raised this to 36%	Require MS/MS confirmation for biomarker-grade lipid IDs
Reis et al., 2021 [57]	Pre-analytical stability study (serum/plasma)	Storage at 4 °C altered lipid levels within 3 days; single freeze–thaw changed LPC and diacylglycerol	Standardise pre-analytical SOPs across all IBD lipidomic studies
Höring et al., 2021 [58]	Isobaric overlap and adduct variability in LC-MS	Co-eluting isobaric species caused up to 70% variation in adduct proportions	Use class-specific isotope-labelled internal standards
Li et al., 2023 [59]	Lipidomics in thiopurine-treated vs. drug-naïve IBD patients	Drug-induced leukopenia produced lipid shifts indistinguishable from disease signal	Use drug-naïve or washout cohorts for biomarker discovery
Salihovic et al., 2024 [9]	Multi-cohort inception-design lipidomics	Most IBD lipidomic studies use 20–50 patient single-centre cohorts; high overfitting risk	Require external validation in ethnically distinct cohorts
Tews et al., 2024 [20]	Biological variable effects on lipoprotein subclass composition	Sex, age, and BMI independently alter lipoprotein subclass levels if unmodelled	Include sex, age, and BMI as covariates in all multivariate models
Yang et al., 2022 [48]	Single-arm before-and-after lipidomics in CD patients on EEN	No comparator arm to separate EEN-induced lipid remodelling from remission	Include a comparator arm or stratify by remission status
Bjerrum et al., 2022 [49]; Diab et al., 2025 [29]	Cross-sectional lipidomics in UC and CD	Single-time-point design precludes trajectory assessment or relapse prediction	Design prospective studies with serial sampling aligned to endoscopic outcomes

## Data Availability

No new data were created or analysed in this study. Data sharing is not applicable to this article.

## Abbreviations

The following abbreviations are used in this manuscript:

IBD	Inflammatory Bowel Disease
CD	Crohn’s Disease
UC	Ulcerative Colitis
IBD-U	IBD-unclassified
PSC	Primary Sclerosing Cholangitis
IBS	Irritable Bowel Syndrome
D-IBS	Diarrhoea-predominant IBS
SCFA/SCFAs	Short-Chain Fatty Acid(s)
MCFA	Medium-Chain Fatty Acids
LCFA	Long-Chain Fatty Acids
VLCFA	Very-Long-Chain Fatty Acids
SFA	Saturated Fatty Acids
MUFA	Monounsaturated Fatty Acids
PUFA	Polyunsaturated Fatty Acids
EPA	Eicosapentaenoic Acid
DHA	Docosahexaenoic Acid
SPM/SPMs	Specialised Pro-Resolving Mediator(s)
S1P	Sphingosine-1-phosphate
C1P	Ceramide-1-phosphate
Cer	Ceramide
HexCer	Hexosylceramide
SM	Sphingomyelin
PC	Phosphatidylcholine
PE	Phosphatidylethanolamine
PS	Phosphatidylserine
PI	Phosphatidylinositol
LPC	Lysophosphatidylcholine
LPE	Lysophosphatidylethanolamine
LPA	Lysophosphatidic Acid
LacCer	Lactosylceramide
PGE_2_	Prostaglandin E2
PGD_2_	Prostaglandin D2
TXB_2_	Thromboxane B2
HETE	Hydroxyeicosatetraenoic acid
LXA_4_/LXB_4_	Lipoxin A4/B4
RvE1/RvD1/RvD2	Resolvins
LTB_4_	Leukotriene B4
CRP	C-reactive protein
IL	Interleukin (es. IL-10, IL-22)
TNF-α	Tumour Necrosis Factor alpha
NF-κB	Nuclear Factor kappa B
Treg	Regulatory T cells
ILC	Innate Lymphoid Cells
AhR	Aryl hydrocarbon receptor
HIF-1α	Hypoxia-inducible factor 1-alpha
HDL-C	High-Density Lipoprotein Cholesterol
LDL	Low-Density Lipoprotein
VLDL	Very Low-Density Lipoprotein
ApoA1/ApoA2	Apolipoproteins
CETP	Cholesteryl Ester Transfer Protein
LC-MS/MS	Liquid Chromatography–Tandem Mass Spectrometry
GC-MS	Gas Chromatography–Mass Spectrometry
MALDI-MSI	Matrix-Assisted Laser Desorption/Ionisation Mass Spectrometry Imaging
DIMS	Direct Infusion Mass Spectrometry
NMR	Nuclear Magnetic Resonance
UPLC-QTOF-MS	Ultra-Performance Liquid Chromatography Quadrupole Time-of-Flight Mass Spectrometry
RCT	Randomised Controlled Trial
AUROC	Area Under the Receiver Operating Characteristic curve
CDAI	Crohn’s Disease Activity Index
SOP	Standard Operating Procedure
IS	Internal Standard
ER	Endoplasmic Reticulum
FAO	Fatty Acid Oxidation
